# Oculomotor, vestibular, reaction time, and cognitive tests as objective measures of neural deficits in patients post COVID-19 infection

**DOI:** 10.3389/fneur.2022.919596

**Published:** 2022-09-12

**Authors:** Kevin M. Kelly, R. Anghinah, A. Kullmann, R. C. Ashmore, A. S. Synowiec, L. C. Gibson, L. Manfrinati, A. de Araújo, R. R. Spera, S. M. D. Brucki, R. L. Tuma, A. Braverman, A. Kiderman

**Affiliations:** ^1^Neurology Department, Allegheny Health Network, Pittsburgh, PA, United States; ^2^Neurology Department at Clinical Hospital of Medical School of University of Sáo Paulo, Sáo Paulo, Brazil; ^3^Medical Department of Athletes Union of Sáo Paulo, Sáo Paulo, Brazil; ^4^Neurolign USA LLC, A Subsidiary of Neurolign Technologies Inc., Pittsburgh, PA, United States

**Keywords:** oculomotor, COVID-19, biomarker, eye-tracking, Neurobehavioral Symptom Inventory (NSI)

## Abstract

**Objective:**

An alarming proportion (>30%) of patients affected by SARS-CoV-2 (COVID-19) continue to experience neurological symptoms, including headache, dizziness, smell and/or taste abnormalities, and impaired consciousness (brain fog), after recovery from the acute infection. These symptoms are self-reported and vary from patient to patient, making it difficult to accurately diagnose and initiate a proper treatment course. Objective measures to identify and quantify neural deficits underlying the symptom profiles are lacking. This study tested the hypothesis that oculomotor, vestibular, reaction time, and cognitive (OVRT-C) testing using eye-tracking can objectively identify and measure functional neural deficits post COVID-19 infection.

**Methods:**

Subjects diagnosed with COVID-19 (*n* = 77) were tested post-infection with a battery of 20 OVRT-C tests delivered on a portable eye-tracking device (Neurolign Dx100). Data from 14 tests were compared to previously collected normative data from subjects with similar demographics. Post-COVID subjects were also administered the Neurobehavioral Symptom Inventory (NSI) for symptom evaluation.

**Results:**

A significant percentage of post COVID-19 patients (up to 86%) scored outside the norms in 12 out of 14 tests, with smooth pursuit and optokinetic responses being most severely affected. A multivariate model constructed using stepwise logistic regression identified 6 metrics as significant indicators of post-COVID patients. The area under the receiver operating characteristic curve (AUC) was 0.89, the estimated specificity was 98% (with cutoff value of 0.5) and the sensitivity was 88%. There were moderate but significant correlations between NSI domain key variables and OVRT-C tests.

**Conclusions:**

This study demonstrates the feasibility of OVRT-C testing to provide objective measures of neural deficits in people recovering from COVID-19 infection. Such testing may serve as an efficient tool for identifying hidden neurological deficits post COVID-19, screening patients at risk of developing long COVID, and may help guide rehabilitation and treatment strategies.

## Introduction

Two years into the global SARS-CoV-2 (COVID-19) pandemic, researchers and clinicians have been confronted with the long-term effects of the infection. Consistent with other coronaviruses, there is growing evidence the virus can cause damage to the central nervous system in addition to its well-known respiratory complications [(1, 2), see (3) and (4) for reviews]. This evidence became apparent early in the pandemic when clinicians worldwide reported neurological symptoms in a high proportion of infected patients; for example, up to 80% of hospitalized patients experience neurological manifestations at some point during disease progression (5, 6). Headache, dizziness, smell and/or taste abnormalities, and impaired consciousness (or “brain fog”) are the most reported neurological symptoms of COVID-19 (7).

These symptoms may be consequences of glial and neural cells in the nervous system expressing angiotensin converting enzyme-2 (ACE-2) receptors, through which the virus enters the body, rendering the brain directly vulnerable to the virus during infection (8, 9). Indeed, viral proteins and RNA have been found in the central nervous system (CNS) of deceased patients at autopsy, in additional to neuropathological findings such as notable inflammation of the brainsteam and damage to the medulla [(10, 11); but see (12) for cases of neuropathological findings in deceased patients with no viral RNA in cortical tissue]. COVID-19 infection can also affect the brain indirectly by causing encephalitis, thrombosis, stroke, coma, and/or hypoxia (6, 13–15). An emerging theme across multiple studies is that immune system hyperactivation may underly much of COVID-19's neurological effects [see (3) and (16) for reviews].

Data have shown that adverse neurological symptoms persist in a significant proportion of patients following recovery, a condition colloquially referred to as “long COVID” and categorized under names such as “post-acute sequelae of COVID-19 syndrome (PASC),” among others. Some reports put the incidence of PASC at approximately one third of infected patients (17–19). In patients that did not require hospitalization, the estimates range from 10–35%; however, in patients that required hospitalization that estimate rises to approximately 85% (20). In one study, the most common enduring symptoms included fatigue, headache, dyspnea, and anosmia (21). Another survey of 3,762 patients, taken 28 days to several months after presumed or confirmed COVID-19 infection, verified these symptoms but also revealed cognitive impairment, memory loss, sleep disruption, and dizziness/vertigo/balance issues as frequent neuropsychiatric symptoms (22).

Much like acute COVID-19 infection, these neurological symptoms of PASC are highly variable among patients, presenting a challenge for precise classification of long COVID as a specific disorder. Additionally, it is possible that neurological damage caused by COVID-19 infection may manifest in subtler neurological deficits of which patients may be unaware. Indeed, as a group, even recovered patients reporting no ongoing COVID-19 symptoms have significant cognitive deficits when compared to healthy controls (23).

As with other fatigue-related syndromes that are identified primarily by patient self-report of symptoms (e.g., chronic fatigue syndrome), objective measures add valuable quantification that otherwise very important symptom profiles lack. To address the need for such measures in COVID-19 survivors, we investigated how recovered patients perform on a comprehensive battery of oculomotor, vestibular, reaction time, and cognitive (OVRT-C) tests, as assessed by high-resolution video oculography (VOG). OVRT-C test metrics are quantifiable and objective proxies and biomarkers of neural function that have been used or investigated in patients with neurological damage due to injury or disease. For example, OVRT-C-based classification models can reliably identify concussion in high school athletes (24), and similar methods have shown promising results in adults with concussion (25). Abnormal oculomotor behaviors have also been proposed as potential biomarkers for several diseases, such as impaired convergence in Parkinson's disease (26), visual paired comparison task performance in patients with mild cognitive impairment (MCI); (27), atypical saccades in preclinical and early-stage Huntington's disease (28, 29), and multiple oculomotor deficits in multiple sclerosis [see (30) for review]. Therefore, OVRT-C testing using eye-tracking technology provides a unique tool to assess overall neural function, while objectively homing in on specific abnormalities that may be affected by COVID-19 infection.

Two recent studies tested the hypotheses that traditional oculomotor and vestibular tests, respectively, can identify neurological abnormalities in recovered COVID-19 (post-COVID) patients. Results of an eye-tracking study suggested that 9 COVID-19-recovered patients exhibited abnormal latency for centrally directed saccades, impaired memory-guided saccades, and increased latencies for antisaccades (31). In the second study, recovered patients had more asymmetries in their vestibular responses, particularly in ocular and cervical vestibular myogenic evoked potentials (oVEMP and cVEMP), and showed a low gain in the video head impulse test (vHIT), suggesting the audio-vestibular system can be damaged, at least temporarily, by COVID-19 infection (32). These reports are consistent with a longitudinal study that detected brain structure changes in previously infected patients compared to controls, including tissue damage in areas associated with the primary olfactory cortex and a greater decrease in overall brain size (33).

In this study we extended these findings, adding measures of pursuit tracking, vergence, optokinetic nystagmus, gaze stability, and reaction time to the investigation of VOG performance in recovered COVID-19 patients. We administered a battery of OVRT-C tests to 77 COVID-19 patients following their recovery from the acute infection stage of the illness. These tests allowed us to identify specific OVRT-C metrics—and the overall *pattern* of deficits—that are uniquely characteristic to post-COVID patients. During their visit, participants also completed the Neurobehavioral Symptom Inventory (NSI) to assess their current neurological symptoms, and to determine whether any of these symptoms were associated with specific OVRT-C metrics.

## Materials and methods

### Participants

All research activities were conducted according to the principles expressed in the Declaration of Helsinki and were approved by the Institutional Review Boards (IRB) at the sites where research was performed. Participants were 77 adults aged 18–45 years of age (see demographics in Table 1) from three groups recruited from three testing sites: (a) Allegheny Health Network, Pittsburgh, PA, USA (IRB#2020-367), *n* = 20; (b) University of São Paulo School of Medicine, São Paulo, Brazil, *n* = 19 and (c) São Paulo School Athletes Union, Brazil *n* = 38 (IRB#33787520.4.0000.0068). Subjects tested at the São Paulo School Athletes Union were primarily amateur and professional athletes (*n* = 38; Cohort 2), while subjects from the Allegheny Health Network and University of São Paulo School of Medicine were patients with no specific athletic categorization (*n* = 39; Cohort 1).

**Table 1:** Demographics of included participants at each testing site and overall.

**Testing sites**	**Participants**	**N**	**Mean age**	**Sex**
			**(years ±SD)**	**(M/F)**
*post-COVID*				
Allegheny Health Network, Pittsburgh, PA	Non-athlete	20	35.9 ± 6.36	9/11
University of São Paulo School of Medicine, Brazil	Non-athlete	19	37.3 ± 11.85	9/10
São Paulo School Athletes Union, Brazil	Athletes	38	29.7 ± 10.15	23/15
Total		77	32.4 ± 9.72	40/37
*Normative data*				
Naval Medical Center San Diego, San Diego, CA	Civilians/Military	48	28.0 ± 6.19	34/14
Madigan Army Medical Center, Fort Lewis, W-ton	Civilians/Military	252	27.3 ± 6.30	172/81
Total		300	27.4 ± 6.28	205/95

M, male; F, female; SD, standard deviation; N, number of participants. Post-COVID patients were divided into two cohorts. Cohort 1 consisted of non-athletes (AHN and USP testing sites), while Cohort 2 came from the SPSAU site, where primarily professional and amateur athletes were tested. AHN, Allegheny Health Network, Pittsburgh, PA. USP, University of São Paulo School of Medicine, São Paulo, Brazil. SPSAU, São Paulo School Athletes Union, São Paulo, Brazil. The normative data participants were tested at two sites, and were representative of a healthy population, collected prior to the COVID-19 pandemic.

All participants reported having been diagnosed with and recovered from the acute COVID-19 infection but experienced persistent and function-impairing symptoms at least 4 weeks after initial infection. None of the subjects reported being hospitalized due to their COVID-19 infection. Each participant gave informed consent prior to testing. Exclusion criteria included those conditions and/or diseases that could influence the oculomotor or vestibular systems (e.g., a history of brain injury, vestibular disorders, blurred vision, or seizures). Special populations including children under 18 years of age, pregnant women, and individuals with diminished capacity (e.g., intellectual disability, dementia) were also excluded from this study. All three sites utilized the same inclusion/exclusion criteria, which are described in Table 2.

**Table 2:** Inclusion and exclusion criteria.

**Inclusion criteria**	**Adults males and females aged 18–45 who have tested positive for SARS-CoV-2 and since recovered**.
Exclusion criteria	1. Pregnancy, as documented by Last Menstrual Period at study visits. Pregnancy is exclusionary because rapid movements are not recommended for pregnant women, and the device will not be intended for use in pregnancy.
	2. Brain injury: a. resulting from a penetrating wound to the head, neck, face or brain (to include gunshot wounds) b. Persons with a previous history of multiple mTBIs in the past. 3. Implants: persons implanted with an electrical and/or neurostimulator device, including but not limited to cardiac pacemaker, defibrillator, vagal neurostimulator, deep brain stimulator, spinal stimulator, bone growth stimulator, or cochlear implant, metal cervical spine hardware. 4. Repeated history of syncope. 5. Presence of severe aphasia. 6. History of chronic vestibular diseases (including Ménière's disease, acute labyrinthitis, vestibular migraine, vestibular neuritis, vestibular schwannoma, sudden sensorineural hearing loss), vestibular dysfunction, previous episode of acute unilateral vestibulopathy or prolonged vertigo. 7. History of prior acute central vestibular lesion. 8. History of prior acute central vestibular lesion. 9. Acute or chronic disease of middle ear (infections, otitis). 10. Past or concomitant treatment with ototoxic chemotherapy. 11. Past history of seizures or convulsions. 12. History of neuropsychiatric disorders antedating the head injury (e.g., hypochondriasis, major depression, schizophrenia). 13. Diagnosed with a learning disability, attention deficit hyperactivity disorder (ADHD), or other neurocognitive or neurobehavioral disorder of childhood (e.g., autism spectrum disorder, major depression, bipolar disorders). 14. Documented neurodegenerative disorders (Multiple sclerosis, Parkinson's, Alzheimer's, Huntington). 15. Neurological disorders including stroke, brainstem or cerebellar dysfunction within the last 3 months. 16. Cerebrovascular disorders. 17. Systemic disorders: e.g., chronic renal failure, cirrhosis of the liver, diabetes, hypertension etc. Version: March 22, 2021 vii. 18. Previous contraindicating surgeries at the discretion of the study physicians or audiologists. 19. Aminoglycosides in the past 6 months given *via* systemic or transtympanic administration.
	20. Concomitant treatment with any of the followings within the last 24 h prior to testing if more than 2 doses have been taken: a. Antihistamines: e.g., diphenhydramine, cyclizine, dimenhydrinate, meclizine, hydroxyzine, promethazine, b. For ADHD and narcolepsy: e.g., Concerta, Daytrana, Methylin, Ritalin, Ritalin LA, Metadate ER, Aptensio XR, Cotempla XR-ODT, QuilliChew ER, and Quillivant XR c. For schizophrenia and other mental diseases: e.g., Phenothiazines d. Specific antibiotics: e.g., ethambutol, gentamycin e. Anticonvulsant medications: e.g., topiramate. 21. Currently suffering from dehydration. 22. History or suspicion of substance abuse or addiction.

For the control population we used previously collected and published data (34, 35).

### Battery of tests

Subjects were administered a battery of 20 OVRT-C tests (see Table 3 for a list of tests with output metrics and length for each test). Of these, the Gaze Horizontal, Spontaneous Nystagmus, Subjective Visual Vertical, and Subjective Visual Horizontal tests assess vestibular function. All stimuli were presented at high contrast on a neutral background with no distracting patterns. Tests were presented consecutively and ranged from 7 to 90 seconds in duration, for a total testing time of approximately 15 min. Subjects were seated comfortably adjacent to the test administrator throughout testing and their heads were not stabilized.

**Table 3:** Tests and metrics in the OVRT-C testing protocol.

	**Tests and parameters**	**Metrics measured for each test**	**Test length (sec)**
1	Auditory Reaction Time (ART): 20–25 sound stimuli are presented with a random timing. The subject is directed to signal their recognition by pressing a button.	Latency (msec) = time difference from stimulus presentation until button is pressed	15
2	Visual Reaction Time (VRT): 20–25 light stimuli are presented in the center of the screen, with a random timing. The subject is directed to signal their recognition by pressing a button.	Latency (msec) = time difference from stimulus presentation until button is pressed	15
3	Subjective Visual *-* Vertical (SVV): subject is presented with a non-vertical line and by using the left and right buttons on the handheld control box, orient the line to the vertical (upright) position, and then press the accept button on the control box.	Mean error (deg) = difference between subject's orientation angle and true vertical. Data are presented as a mean of errors of all measurements.	15
4	Self-Paced Saccade (SPS): subject is required to do saccades between two dots for 20–30 s.	a) Number of saccades = how many saccades are performed during the test time	21
		b) Eye velocity consistency – eye velocity for left/right eye during tests; measures consistency and fatigue	
		c) Interval consistency between saccades – time between the saccades; measures consistency and fatigue	
5	Saccade and Reaction Time (SRT): 30 visual saccadic stimuli are randomly projected every 1 to 2 s with a displacement of −30 to + 30 degrees. The subjects are directed to gaze at the red dot (saccadic stimulus) and then press either the left or right button to record whether the stimulus was projected to the right or to the left.	a) Latency (s)	45
		b) Accuracy (%)	
		c) Final Accuracy (%)	
		Motor reaction time variables: d) Latency means (s) – for Left Button = time difference from stimulus presentation until the left button is pressed Latency means (s) – for Right Button= time difference from stimulus presentation until the right button is pressed.	
6	Saccade *-* Random, Horizontal (SH): subject follows a dot displayed 30 times at pseudo-randomly distributed times (between 1 to 2 s) and pseudo-random displacements on either a horizontal plane (−30 to +30 degrees).	a) Latency (s) = time from stimulus presentation until a saccade is initiated. Data are presented as an average of all saccade onset latencies.	22
		b) Accuracy (%) = difference between eye position and stimulus position for the main saccade, expressed in percentage relative to stimulus position. Data are presented as an average of all main saccade accuracies.	
		c) Final Accuracy (%) = difference between eye position and stimulus position for the final position, including corrective saccades, expressed in percentage relative to stimulus position. Data are presented as an average of all saccade accuracies.	
		d) Area Under Main Sequence Fit (AUF) (deg^2^/sec). Eye velocity is plotted as a function of saccade displacement and fitted with an exponential function. To evaluate the overall velocity and amplitude relationship, the software computes the area under the curve, out to 30 degrees of eye displacement = AUF.	
		e) Peak velocity = eye velocity corresponding to each eye displacement in response to a stimulus displacement	
7	Saccade *-* Random, Vertical (SV): subject follows a dot displayed 30 times at pseudo-randomly distributed times (between 1 to 2 s) and pseudo-random displacements on either a vertical plane (−20 to +20 degrees).	Same variables as above.	22
8	Smooth Pursuit: subject follows a dot as it displaced (moves) sinusoidally horizontally then vertically at different speeds: *S*mooth Pursuit – Horizontal (SPH) 0.1 Hz, 3 cycles; 0.75 Hz 6 cycles	a) Velocity Gain = ratio between the slow phase component of eye velocity and pursuit tracker stimuli. Data are averaged for the leftward and rightward moving stimuli.	40
		b) Asymmetry = Velocity Gain Asymmetry; represents the difference between gain calculated for leftward and rightward moving stimuli – see calculations below the table	
		c) Position Gain = ratio between the ratio between the slow phase component of eye velocity and pursuit tracker stimuli - see calculations below	
		d) Saccadic component (%) = percentage of eye movement spent on a saccadic movement vs. pursuit movement	
		e) Initiation latency (msec) = time from stimulus presentation until a smooth pursuit movement is initiated.	
9	Smooth Pursuit *-* Vertical (SPV) 0.1 Hz, 3 cycles; 0.75 Hz 6 cycles	Same as above.	40
10	Vergence Pursuit (VP): the subject is required to follow a light stimulus that moves towards and away from the subject in a smooth pursuit pattern, 0.1 Hz 3 cycles	a) Left/Right eye gain = how well the subject tracks the stimulus, calculated for each eye	30
		b) Left-right eyes correlation = how well left-right eye correlate between each anther	
		c) Saccadic components (%) = percentage of eye movement spent on a saccadic movement versus pursuit movement	
11	Vergence Step (VS): the subject is required to follow a light stimulus that moves towards and away from the subject in a saccade (step) pattern (9 cycles).	a) Left/Right eye inward and outward time constant = how well the subject tracks the stimulus, calculated for each eye	20
		b) Left/Right eyes correlation = how well left-right eye correlate between each other in inward and outward directions	
		c) Saccadic components (%) = percentage of eye movement spent on a saccadic movement	
12	Optokinetic (OKN) 20 deg/s: subjects see a field of dots moving on the display first to the right, then to the left, with eye tracking throughout the test with a velocity of 40 deg/s. Each test consists of a stimulus rotating for 10 s clockwise (CW) and then 10 s counterclockwise (CCW), with 3 s of rest between CW and CCW rotation.	a) Average eye velocity CW and CCW (deg/sec) = eye velocity during the slow phase of nystagmus for stimuli moving in clockwise (CW) and counterclockwise (CCW) direction	25
		b) Gain = ratio between average eye slow phase velocity and stimulus for CW and CCW segments	
		c) Gain Asymmetry (%) = represents the difference between gain calculated for CW and CCW segments - see calculations below	
		d) Area Under Main Sequence Fit (AUF) (deg2/sec). Fast phase of OKN nystagmus beats is plotted as a function of the beats length and fitted with an exponential function. To evaluate the overall velocity and amplitude relationship, the software computes the area under the curve = AUF for CW and CCW stimulus movement.	
		e) Normalized OKN CW velocity gain (normalized at 20 deg/sec)	
		f) Normalized OKN CCW velocity gain (normalized at 20 deg/sec)	
13	Optokinetic (OKN) 60 deg/s	Same as above.	25
14	Predictive Saccades (PS): subject is directed to follow a dot as it is displayed. Subject is presented with 6 pseudo-random saccade stimuli followed by 21 mirrored saccade stimuli with repeated displacement +/-10 degrees, horizontal, at a constant time interval of 0.65 s.	First predicted, total number predicted saccades, % of predicted saccades	18
15	Antisaccades (AS): subject is required to fixate on a central target for 1.5 to 2.5 s. Then the subject is presented with a peripheral target. Subject is required to generate an eye movement in the same distance as the target displacement, but in the exact opposite direction. There are 33 anti-saccades with time between saccades randomly selected from 1 to 2 s and random displacement between −30 to + 30 degrees.	Error Rate (%) = percentage of pro-saccade errors, i.e., where the subject looks toward rather than away from the stimulus	22
16	Spontaneous Nystagmus (SN) – subject fixates a light stimulus placed on the center of the screen for 10 s. The light is turned off and the subject is required to continue to fixate at the spot where the light was for 15 s.	Direction and velocity of nystagmus beats and number of square wave jerks for horizontal and vertical nystagmus during fixation and in the dark	25
17	Gaze Horizontal (GH) -subject fixates a light stimulus placed on 15 deg from the center to the left and then to the right of the screen for 10 s. The light is turned off and the subject is required to continue to fixate at the spot where the light was for 15 s.	Direction and velocity of nystagmus beats and number of square wave jerks for horizontal and vertical nystagmus during fixation and in the dark with gaze to the left and right	50
18	Subjective Visual – Horizontal (SVH): subject is presented with a non-horizontal line and by using the left and right buttons on the handheld control box, orient the line to the horizontal (straight across) position, and then press accept button on the control box.	Mean error (deg) = difference between subject's orientation angle and true horizontal. Data are presented as a mean of errors of all measurements.	15
**Testing time**			~8 min
**Set-up time**			**2 min**
**Test Instructions**			**2 min**
**Total time**			**12 min**

### Hardware and software

OVRT-C tests were delivered *via* the Neurolign Dx 100 (formerly known as I-Portal Portable Assessment System - Nystagmograph; I-PAS), an FDA-cleared eye-tracking device manufactured by Neurolign USA, LLC (formerly known as Neuro Kinetics, Inc.; Pittsburgh, PA). The Neurolign Dx 100 is a portable and compact head-mounted virtual reality goggle set equipped with high-speed digital infrared cameras (940 nm, sampling rate of 100 frames/second) that capture high-resolution images of eye movements in response to light and auditory stimuli. A hand-held apparatus with response buttons recorded participants' reaction times and/or responses during tests that require manual input (i.e., subjective visual vertical/horizontal, auditory reaction time). Data were collected using Neurolign's I-Portal software, which captures time stamps (necessary for synchronization) and analyzes digital images of the eye to record horizontal and vertical eye movement data. Proprietary VEST™ software was utilized to operate the hardware, create and edit stimulus parameters, integrate I-Portal eye-tracking results, and analyze acquired data to generate a comprehensive set of desired metrics.

### Treatment of artifacts and outlying samples

Data were calibrated for position by comparing eye movements to fixation locations that had a known displacement. VEST™ software identified artifacts such as blinks, recording noise, and any temporary failures in eye tracking. Along with other possible artifacts, such as shifting of goggles during testing, incorrect responses, and/or responses not related to the task, all artifacts were evaluated manually to ensure discrimination of eye movement signals from recording noise. In some cases, manual analysis was also necessary to isolate saccadic activity from pursuit activity. VEST™ software automatically reports data validity, i.e., the percentage of acceptable data available from which to calculate results. The software alerted the test administrator if data validity was below 60%, in which case VEST™ only analyzed data from a single eye as long as that eye was above the 60% validity criterion. If validity for both eyes was below 60%, data from that particular test were discarded. For some subjects, results from specific OVRT-C tests were removed from analysis when the data quality was inadequate for accurate measurement or created analytic errors.

### Data analysis

Data acquired for each OVRT-C test were reviewed to ensure completion and validity, and analyzed using VEST™ software.

For each completed and validated test for every subject, measures of OVRT-C performance were collected from the VEST™ software. These measures corresponded to the “Metrics measured for each test” in Table 3, and “Metrics” in Table 4. For a given subject, each of their collected metric measurements were compared to the normative range for that metric in a clinical (FDA-approved) normative database (34, 35). We then determined the number of subjects whose measures fell within vs. outside the normative range for that metric. These are reported as percentage (%) abnormalities for each variable. Since the normative ranges were based on a 95^th^ percentile ranking (95% of the normative database subjects were within each range), the expected percentage outside each range (% Abnormalities) would be 5% for an unaffected subject population. Our results are therefore meant to be interpreted relative to this expected 5%.

**Table 4:** Comparison of the two COVID-19 cohorts.

**Test**	**Metrics**	***non-Athletes***			***Athletes***			***p*-value1^1^**
		***N***	**Mean**	**SD**	***N***	**Mean**	***SD***	**(*Mean Diff*)**
Auditory RT	Mean latency	38	235	90	38	210	34	0.116
Visual RT	Mean latency	38	250	50	38	241	36	0.388
Subjective Visual Vertical	Overall error mean	39	0.16	1.82	38	−1.34	2.02	**0.001**
Self-paced Saccades	Saccades per second	38	2.00	0.81	38	2.30	0.62	0.073
	Position error degrees mean	38	2.07	0.91	38	1.99	0.97	0.717
Saccade and RT	Rightward latency means	38	0.21	0.03	37	0.21	0.06	0.913
	Leftward latency means	38	0.20	0.05	37	0.19	0.06	0.336
	Motor resp R button latency means Motor resp right button latency mean	38	0.52	0.16	38	0.51	0.14	0.700
	Motor resp L button latency means	38	0.53	0.15	38	0.47	0.15	0.074
	Latency grand mean	38	0.21	0.03	37	0.20	0.05	0.658
	Accuracy grand mean	38	89.6	12.3	37	90.0	16.3	0.896
	Final accuracy grand mean	38	96.3	13.4	37	95.5	16.4	0.825
Saccades Horizontal	Latency grand mean	39	0.20	0.03	38	0.19	0.02	**0.003**
	Accuracy grand mean	39	92.4	11.1	38	91.4	6.4	0.608
	Final accuracy grand mean	39	98.8	11.3	38	96.1	5.0	0.186
	(RR) Accuracy % of undershoot	39	21.1	19.6	38	23.9	18.4	0.522
	(LL) Accuracy % of overshoot	39	7.8	11.8	38	8.8	11.5	0.720
	(RR) Final acc. % undershoot	39	20.2	16.6	38	22.2	16.8	0.605
	(RR) Final acc. % overshoot	39	9.7	13.2	38	8.8	14.9	0.784
Saccades Vertical	Latency grand mean	39	0.21	0.03	38	0.19	0.02	**0.001**
	Accuracy grand mean	39	92.8	12.2	38	94.4	10.8	0.563
	Final accuracy grand mean	39	99.1	11.4	38	96.9	10.0	0.372
	(LD) Accuracy % of undershoot	39	16.7	16.7	38	13.5	16.1	0.392
	(LD) Accuracy % of overshoot	39	12.9	15.1	38	18.4	18.2	0.153
	(LD) Final acc. % of undershoot	39	14.5	19.4	38	14.1	16.4	0.928
	(RD) Final acc. % of undershoot	39	13.0	18.9	38	16.5	16.2	0.381
Smooth Pursuit Horizontal, 0.1 Hz	Velocity gain rightward	39	0.9	0.1	38	1.0	0.1	0.379
	Velocity gain leftward	39	1.0	0.1	38	0.9	0.1	0.408
	Velocity saccade, %	39	29.3	20.1	38	26.0	11.6	0.366
	Position gain	39	1.01	0.02	38	1.00	0.03	0.070
	Initiation latency msec	39	268.5	72.1	38	250.2	88.6	0.325
Smooth Pursuit Horizontal, 0.75 Hz	Velocity gain rightward	39	0.9	0.2	38	0.9	0.2	0.460
	Velocity gain leftward	39	0.9	0.2	38	0.9	0.1	0.509
	Velocity saccade, %	39	32.5	16.2	38	30.2	16.6	0.545
	Position gain	39	1.01	0.07	38	1.06	0.13	0.063
	Initiation latency msec	39	233.2	50.1	38	245.3	57.2	0.329
Smooth Pursuit Vertical, 0.1 Hz	Velocity gain up	39	0.89	0.18	38	0.94	0.09	0.079
	Velocity gain down	39	0.92	0.11	38	0.93	0.10	0.728
	Velocity saccade, %	39	30.3	17.9	38	24.9	14.9	0.158
	Position gain	39	1.06	0.21	38	1.02	0.06	0.311
	Initiation latency msec	39	257.1	77.7	37	269.9	103.0	0.542
Smooth Pursuit Vertical, 0.75 Hz	Velocity gain up	39	0.82	0.22	38	0.84	0.21	0.639
	Velocity gain down	39	0.69	0.24	38	0.77	0.21	0.161
	Velocity saccade, %	39	38.1	17.3	38	36.0	15.8	0.592
	Position gain	39	1.04	0.15	38	1.06	0.17	0.670
	Initiation latency msec	39	207.8	58.7	38	205.9	37.6	0.866
Vergence Pursuit	Left eye position gain	38	0.79	0.19	38	0.86	0.16	0.107
	Right eye position gain	38	0.79	0.22	38	0.83	0.22	0.418
	Eye correlation inward	38	−0.5	0.8	38	−0.7	0.5	0.184
	Eye correlation outward	38	−0.51	0.72	38	−0.65	0.55	0.350
Vergence Step	Mean inward correlation	38	−0.20	0.73	38	−0.51	0.61	**0.048**
	Mean outward correlation	38	−0.07	0.61	38	−0.17	0.57	0.477
Optokinetic Nystagmus, 20 deg/s	Average gain	38	0.75	0.17	38	0.67	0.24	0.073
	Asymmetry	38	6.8	18.5	38	8.5	19.9	0.712
	CW Area under fit 30	36	8,268	1,974	37	7,797	2,215	0.340
	CCW Area under fit 30	38	−8,407	1,464	38	−7,144	1,809	**0.001**
	Mean area under fit 30	36	8,290	1,554	37	7,485	1,828	**0.046**
Optokinetic Nystagmus, 60 deg/s	Average gain	38	0.35	0.17	38	0.26	0.18	**0.031**
	Asymmetry	38	3.57	26.73	38	−6.06	28.56	0.133
	CW Area under fit 30	37	7,627	1,916	37	7,153	2,072	0.310
	CCW Area under fit 30	37	−7,972	2,195	36	−7,678	2,469	0.592
	Mean area under fit 30	37	7,800	1,813	35	7,504	1,820	0.493
Predictive Saccades	Latency grand mean	39	0.12	0.06	38	0.09	0.05	**0.013**
	(L) % of predicted	39	28.8	22.4	38	38.5	22.5	0.060
	(R) % of predicted	39	28.8	22.0	38	37.5	22.8	0.092
Antisaccades	Overall prosaccade % error	38	42.2	29.0	38	36.0	28.8	0.354
	Accuracy grand mean	38	173	96	38	156	67	0.387
	Leftward prosaccade % error	38	39.6	31.6	38	30.0	30.0	0.576

1 A p-value from a two-sample (mean difference) t-test that determines whether the two independent samples come from distributions with equal means by assuming unknown and unequal variances. The p-value was calculated under a two-tail hypothesis. For simplicity, RT refers to reaction time.

**Figure 1:**
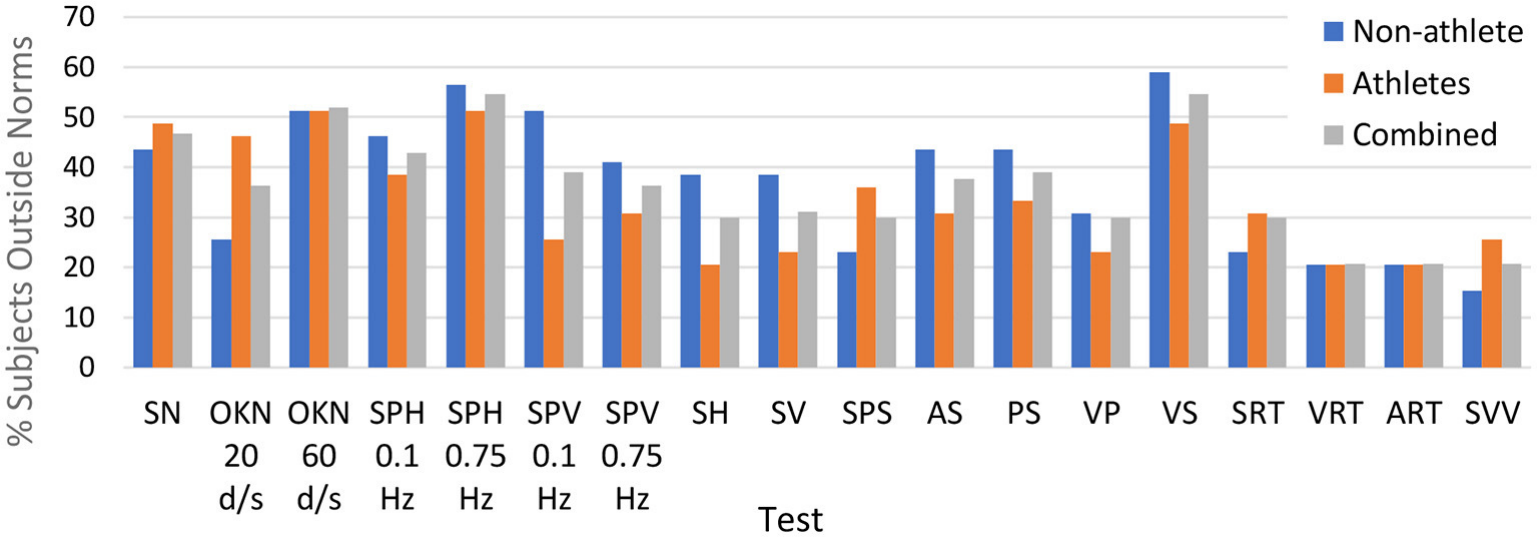
Percentage of subjects in each cohort that had one or more metrics outside normative ranges for each of the tests in our testing protocol.

In some cases, we report % Abnormalities for a whole test (e.g., Optokinetic Nystagmus at 60 deg/sec, see Figure 1), where the whole test consists of multiple metrics. Nonetheless, testing for the significance of differences was performed per metric, not on a whole-test basis.

A *Two-sample t-test* was used to evaluate the similarity between our two patient cohorts (TTEST2 function in MATLAB). Differences between the observed rate of metric measures that fell outside normative ranges vs. the expected (normative) rate were evaluated using a *One-Proportion Z-test*. The significances of these differences (given in Table 5) were computed by


(1) $$\begin{array}{l} Z_{st}=\frac{|p-P_{0}|-c}{\sqrt{P_{0}\left(1-P_{0}\right)/N}}, \end{array}$$

where *c* = 1/(2*N*) is a correction for continuity and *p* is the estimated proportion of post-COVID participants whose test value is outside the normative 95% reference interval (RI) limits (column %Abn), *N* is the total number of post-COVID participants, and *P*_0_ = 0.05 is an expected proportion. The corresponding *p*-value was calculated under a two-tail hypothesis. Because we are performing multiple comparisons, each targeting essentially the same null hypothesis (the proportion of subjects with measures outside the normative range is *P*_0_), we have adjusted our acceptable significance threshold to α <0.0009 (Bonferroni correction).

**Table 5:** Abnormal rates among post-COVID participants and t-test examine the mean difference between post-COVID and healthy participants.

**Tests**	**Metrics**	**Abnormal rate**						**Two-sample** ***t*****-test**		
		**95% RI Limits**		**post-COVID**				**Healthy**	**post-COVID**	***p*-value (Mean diff)**
		***Lower***	***Upper***	***N***	**Abn**	***%*Abn**	***p*–value**	**Mean**	**Mean**	
Auditory RT	Mean latency	*n/a*	316	76	6	7.9%	0.37092778	234	222	0.184
Visual RT	Mean latency	*n/a*	343	76	4	5.3%	0.87453975	274	246	0.000
Subjective Visual Vertical	Overall error mean	−2.96	2.96	77	14	18.2%	0.00000045	—	−0.58	—
Saccade and RT	Saccades Rightward latency mean	*n/a*	0.29	75	2	2.7%	1.00000000	0.20	0.21	0.321
	Saccades Leftward latency mean	*n/a*	0.29	75	2	2.7%	1.00000000	—	0.20	—
	Motor Right button latency mean	*n/a*	0.65	76	12	15.8%	0.00005064	0.50	0.52	0.250
	Motor Left button latency mean	*n/a*	0.65	76	13	17.1%	0.00000467	—	0.50	—
	Saccade latency grand mean	*n/a*	0.29	75	2	2.7%	1.00000000	—	0.20	—
	Saccades final acc. grand mean	79	106	75	21	28.0%	0.00000000	—	95.9	—
Saccades Horizontal	Area under fit mean	8,239	*n/a*	77	3	3.9%	1.00000000	—	1,0382	—
	Latency grand mean	*n/a*	0.22	77	9	11.7%	0.01503958	0.18	0.19	0.000
	Accuracy grand mean	81	103	77	6	7.8%	0.38826750	92.5	91.9	0.587
	Final acc. grand mean	89	104	77	6	7.8%	0.38826750	96.3	97.5	0.272
	Accuracy % of undershoot	—	—	77	—	—	—	6.6	22.5	0.000
	Final accuracy % of undershoot	—	—	77	—	—	—	2.0	21.2	0.000
Saccades Vertical	Area under fit mean	7,630	*n/a*	77	6	7.8%	0.38826750	9,684	9,597	0.608
	Latency grand mean	*n/a*	0.23	77	11	14.3%	0.00050668	0.19	0.20	0.000
	Accuracy grand mean	75	109	77	10	13.0%	0.00313368	92.7	94	0.517
	Final acc. grand mean	79	107	77	13	16.9%	0.00000610	94.2	98	0.004
	Accuracy % of undershoot	—	—	77	—	—	—	18.6	19.9	0.666
	Final accuracy % of undershoot	—	—	77	—	—	—	8.2	13.9	0.025
Smooth Pursuit Horizontal, 0.1 Hz	Velocity gain rightward	0.78	1.07	77	6	7.8%	0.38826750	0.95	0.95	0.509
	Velocity gain leftward	0.78	1.07	77	4	5.2%	0.85478982	—	0.95	—
	Velocity gain asymmetry	−8.80	7.53	77	8	10.4%	0.05632178	—	−0.27	—
	Velocity saccade, %	*n/a*	35	77	17	22.1%	0.00000000	18.0	27.7	0.000
	Position gain	0.96	1.04	77	10	13.0%	0.00313368	1.00	1.01	0.030
	Initiation latency msec	*n/a*	335	77	13	16.9%	0.00000610	—	259	—
Smooth Pursuit Horizontal, 0.75 Hz	Velocity gain rightward	0.62	1.08	77	12	15.6%	0.00006332	0.95	0.92	0.180
	Velocity gain leftward	0.62	1.08	77	14	18.2%	0.00000045	—	0.92	—
	Velocity gain asymmetry	−8.93	9.00	77	18	23.4%	0.00000000	—	−0.04	—
	Velocity saccade, %	*n/a*	37	77	27	35.1%	0.00000000	15.8	31.4	0.000
	Position gain	0.79	1.10	77	12	15.6%	0.00006332	0.96	1.03	0.000
	Initiation latency msec	*n/a*	252	77	31	40.3%	0.00000000	—	239	—
Smooth Pursuit Vertical, 0.1 Hz	Velocity gains up	0.69	1.07	77	9	11.7%	0.01503958	0.90	0.91	0.335
	Velocity gains down	0.69	1.07	77	7	9.1%	0.16585365	—	0.93	—
	Velocity gain asymmetry	−12.36	11.46	77	15	19.5%	0.00000003	—	−1.02	—
	Velocity saccade, %	*n/a*	32.00	77	26	33.8%	0.00000000	14.1	27.66	0.000
	Position gain	0.95	1.07	77	27	35.1%	0.00000000	0.99	1.04	0.013
	Initiation latency msec	*n/a*	311	76	18	23.7%	0.00000000	—	263.3	—
Smooth Pursuit Vertical, 0.75 Hz	Velocity gains up	0.42	1.09	77	9	11.7%	0.01503958	0.81	0.83	0.533
	Velocity gain down	0.42	1.09	77	11	14.3%	0.00050668	—	0.73	—
	Velocity gain asymmetry	−23.43	29.01	77	17	22.1%	0.00000000	—	6.29	—
	Velocity saccade, %	*n/a*	52	77	16	20.8%	0.00000000	26.9	37.0	0.000
	Position gain	0.73	1.11	77	22	28.6%	0.00000000	0.91	1.05	0.000
	Initiation latency msec	*n/a*	230	77	23	29.9%	0.00000000	—	207	—
Optokinetic Nystagmus, 20 deg/s	Average gain	0.66	0.97	76	29	38.2%	0.00000000	0.86	0.71	0.000
	Asymmetry	−7.66	10.55	76	33	43.4%	0.00000000	—	7.65	—
	CW area under fit 30	5,513	*n/a*	73	10	13.7%	0.00168041	8084	8029	0.838
	CCW area under fit 30	*n/a*	−5,956	76	11	14.5%	0.00042138	−8464	−7775	0.002
	Mean area under fit 30	5,735	*n/a*	73	9	12.3%	0.00919937	—	7882	—
Optokinetic Nystagmus, 60 deg/s	Average gain	0.40	0.90	76	54	71.1%	0.00000000	0.61	0.31	0.000
	Asymmetry	−14.54	18.10	76	36	47.4%	0.00000000	—	−1.24	—
	CW area under fit 30	6,289	*n/a*	74	21	28.4%	0.00000000	8106	7390	0.005
	CCW area under fit 30	*n/a*	−6,235	73	18	24.7%	0.00000000	−8177	−7827	0.222
	Mean area under fit 30	6,262	*n/a*	72	16	22.2%	0.00000000	—	7656	—
Predictive saccades	(L) % of predicted	17	*n/a*	77	24	31.2%	0.00000000	65.3	33.6	0.000
	(R) % of predicted	17	*n/a*	77	24	31.2%	0.00000000	65.4	33.1	0.000
Antisaccades	Overall prosaccade % error	0	50	76	22	28.9%	0.00000000	16.3	39.1	0.000
	Acc. grand mean	—	—	76	—	—	—	108.7	164.6	0.000
	Leftward prosaccade % error	—	—	76	—	—	—	15.2	34.8	0.000

n/a refers to a situation with no upper or lower RI limit.

A *Two-Sample t-test* was used to determine whether there is a difference between the mean (test) value of two independent groups – post-COVID 72–77 subjects and 300 healthy (control) participants. The corresponding *p*-value (mean difference) was calculated under a two-tail hypothesis.

We evaluated the value of our test metrics as predictors of COVID-19 status using a series of univariate and multivariate regression models. A logistic regression model was used to estimate the probability of a binary response (COVID / not COVID) as a function of one or more independent metrics. Models were generated using data from the normative database (300 normative data subjects) and 75 of our 77 subjects. We first fit logistic regression coefficients separately for each of 28 OVRT test metrics (FITGLM function in MATLAB using the method of Maximum Likelihood; see Table 6). A multivariate logistic regression model was generated and tested for its ability to distinguish between post-COVID and healthy control participants (evaluated on training data without a separate validation population). A standard stepwise procedure was used to identify those metrics that best fit the model. To evaluate the diagnostic accuracy, in addition to AUC and Somers' D, sensitivity, specificity and overall accuracy was computed with a logistic function cutoff value of 0.5.

**Table 6:** Univariate logistic regression results across tests and metrics.

**Test**	**Metrics**	***N***	**ML estimates**		**Diag. accuracy**	
			**Estimate**	***p-*value**	**AUC**	**Somers' D**
Saccade and RT	Rightward latency means	375	7.928	0.013	0.58	0.17
	Motor resp R button latency mean	376	1.321	0.185	0.53	0.06
Saccade Horizontal	Latency grand mean	377	31.389	0.000	0.68	0.36
	Accuracy % of undershoot	377	0.070	0.000	0.71	0.42
	Final accuracy % of undershoot	377	0.212	0.000	0.79	0.58
Saccade Vertical	Latency grand mean	377	19.741	0.000	0.61	0.22
	Final accuracy grand mean	377	0.057	0.000	0.58	0.16
	Area under fit 30	377	−0.0002	0.035	0.55	0.11
	Final accuracy % of undershoot	377	0.019	0.007	0.55	0.10
Smooth Pursuit Horizontal, 0.1 Hz	Velocity saccade, %	377	0.060	0.000	0.65	0.29
Smooth Pursuit Horizontal, 0.75 Hz	Velocity saccade, %	377	0.075	0.000	0.73	0.45
Smooth Pursuit Vertical, 0.1 Hz	Velocity saccade, %	377	0.093	0.000	0.71	0.42
Smooth Pursuit Vertical, 0.75 Hz	Velocity saccade, %	377	0.044	0.000	0.64	0.29
Optokinetic Nystagmus, 20 deg/s	Average gain	376	−7.805	0.000	0.67	0.34
	CW area under fit 30	373	−0.00002	0.815	0.52	0.03
	CCW area under fit 30	376	0.00027	0.001	0.60	0.19
Optokinetic Nystagmus, 60 deg/s	Average gain	376	−9.690	0.000	0.82	0.63
	CW area under fit 30	374	−0.00024	0.002	0.59	0.17
	CCW area under fit 30	373	0.00013	0.110	0.55	0.10
Predictive saccades	(L) % of predicted	377	−0.051	0.000	0.77	0.53
	(R) % of predicted	377	−0.053	0.000	0.77	0.54
Antisaccades	Overall prosaccade % error	376	0.054	0.000	0.69	0.38
	Accuracy grand mean	376	0.013	0.000	0.69	0.37
	Leftward prosaccade % error	376	0.039	0.000	0.65	0.30

N represents the total number of subjects up to a maximum of 77 post-COVID patients and 300 subjects from the normative data set. Table entries are estimated values of the regression coefficients (ML estimates) with corresponding p-values, the estimated value of the area under the receiver operating characteristic curve (AUC) statistics and the related Somers' D statistics (= 2 AUC – 1).

### Neurobehavioral symptom inventory

The Neurobehavioral Symptom Inventory (NSI) is a 22-item self-report questionnaire commonly administered to patients following traumatic brain injury (TBI). Each item lists a potential neurological symptom and subjects were asked to rate how much each symptom was disturbing them at time of test on a scale of 0 (none/not at all) to 4 (always/very severe), with a total possible score of 88. A recent factor structure analysis of NSI responses by US military members with mTBI and healthy National Guard members (no mTBI) revealed a 4-factor model was the best fit, grouping responses into 4 neurobehavioral domains: vestibular, somatic, cognitive, and affective (36).

Spearman's rank correlation coefficient (rho) was used to measure the relationship between the test variables and NSI symptoms (CORR function in MATLAB); see **Table 8**.

## Results

### Comparison to normative OVRT-C ranges

In this study, we examined the hypothesis that OVRT-C test responses are reliable neurological biomarkers that can be used in the identification and evaluation of post-COVID/PASC patients. Using a clinical (FDA-approved) database of normative OVRT-C values (34, 35) we tested the specific hypothesis that in this population, multiple OVRT-C measures will fall outside established normative ranges.

For each test in our protocol, we identified which subjects had abnormal (outside normative ranges) responses to one or more variables within that test. Figure 1 displays a summary of these abnormality percentages within Cohort 1 (non-athletes), Cohort 2 (athletes), and overall (pooled data). As the figure shows, OVRT-C abnormalities were distributed relatively evenly between the two cohorts. Some tests presented clear challenges for subjects of either cohort. For instance, over half of Cohort 1 and Cohort 2 had impaired (outside norms) performance on the high-speed optokinetic test.

As both cohorts showed deficits, we first evaluated whether there were differences between these two cohorts (Table 4). We found that many metrics were not significantly different (60 out of 68), with the exception of several metrics: overall mean error in the SVV test, saccade latency in all saccade tests, mean inward correlation in the vergence test, clockwise and mean area under the fit in the OKN 20deg/s test, and average gain in the OKN 60 deg/s test. Based on the lack of significant differences, we pooled the two cohorts to further evaluate the deficits by comparing them to normative data. This allowed us to increase the sample size to 77 for a more robust comparison with the 300 controls included in the normative data.

**Figure 2:**
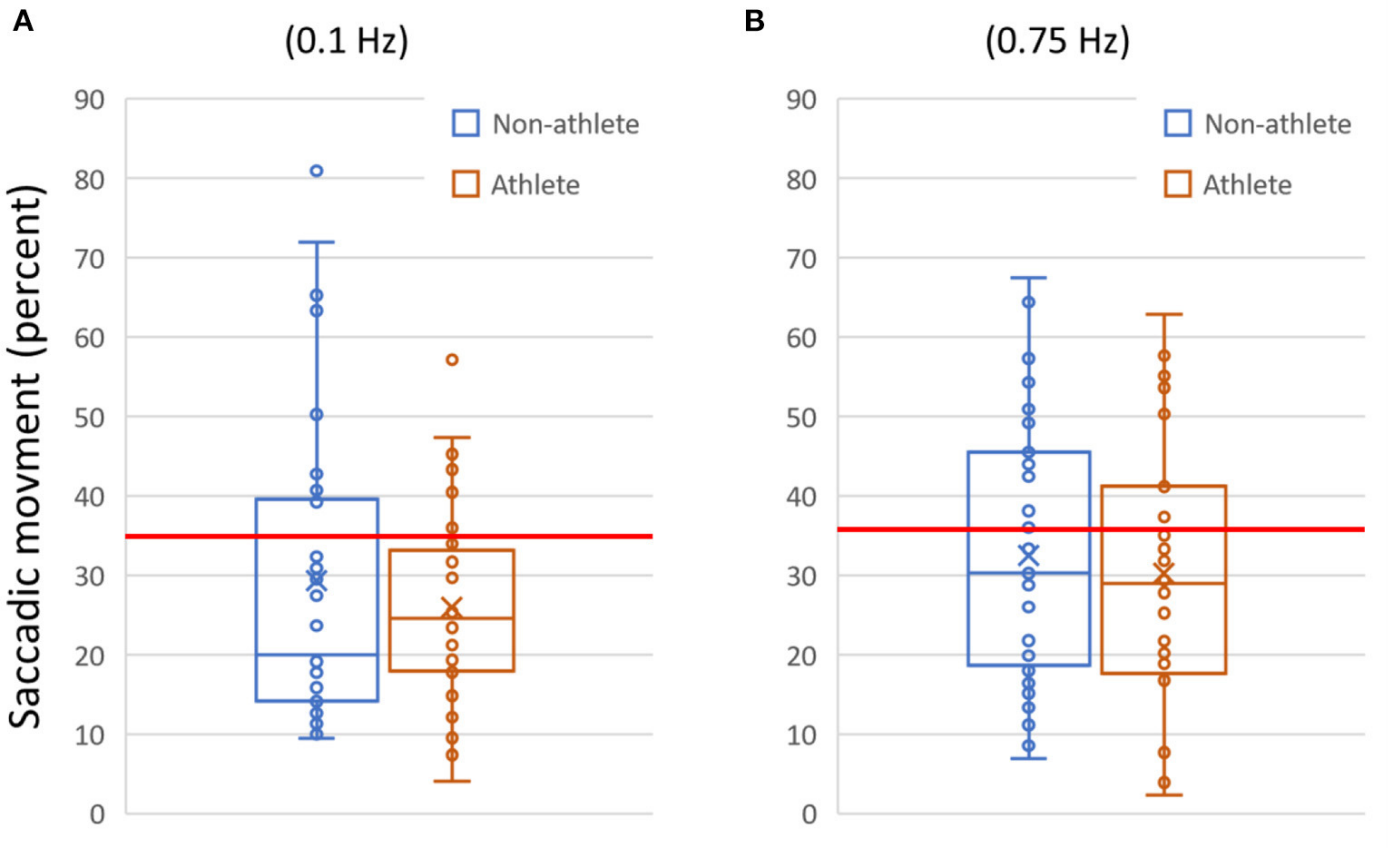
Example data plotted for two variables. **(A)** The amount of saccadic (aberrant non-pursuit) activity measured for each subject during the slow (0.1 Hz) horizontal smooth pursuit test. **(B)** Saccadic activity for the fast (0.75 Hz) horizontal smooth pursuit test. In **(A)** and **(B)**, data are shown as quartile box and whisker plots (with outliers visible). The horizontal line indicates the 95% reference interval for the metric from the normative database (≥35% for 0.1 Hz, ≥37% for 0.75 Hz). In **(A)**, saccadic activity above the threshold was present in 22.1% of participants (*N* = 17/77; 0.1 Hz). In **(B)**, saccadic activity above the threshold was present in 35.1% (*N* = 27/77; 0.75 Hz).

We further examined the individual variables making up each test in more detail in order to highlight where performance difficulties arose (Figure 2, Table 5).

Table 5 provides a list of multiple OVRT-C variables, the observed frequencies of measures outside respective normative ranges for our subject population (pooled data), and an indication of significance based on our acceptance threshold of α <0.0009 (by One-Proportion *Z*-test). This analysis revealed that a significant percentage of subjects scored outside the norms in 12 out of 14 tests and 41 out of 60 metrics. The tests showing the highest percentage of subjects scoring outside the norms for all or many test metrics were smooth pursuit and OKN. The smooth pursuit tracking function revealed multiple significant differences, especially for higher speeds (SPH, 0.75 Hz) and for vertical pursuit tracking (SPV, 0.1 Hz and 0.75 Hz). Figure 2 shows the saccadic component or the amount of saccadic activity during smooth pursuit horizontal at 0.1 Hz and 0.75 Hz for all subjects. Given that the normative thresholds represent the 95^th^ percentile for the normative population, we would expect that approximately 5% of our subjects would exceed each threshold if patients' post-COVID status had no effect on smooth pursuit activity. However, as shown in Figure 2, the number of subjects with measures beyond the respective thresholds was significantly higher in both cases with 22.1% (*N* = 17/77) for 0.1 Hz (*p* < 0.0009 by One-Proportion *Z*-test) and 35.1% (*N* = 27/77) for 0.75 Hz (*p* < 0.0009). These results indicate that a significant number of post-COVID patients have abnormally increased saccadic movement, and therefore less smooth pursuit movement, compared to a normative population.

Optokinetic nystagmus (OKN) responses, which rely on both saccade-like and pursuit-like eye movements, were dramatically affected in our post-COVID subjects (Table 5). Among the OKN responses, 71.1% (*N* = 54/76; *p* < 0.0009) had abnormally low gain for the high speed OKN test, meaning that post-COVID patients were impaired in their ability to generate higher speed nystagmus movements (the slow phase of OKN was significantly slower than the normative velocity).

Notably, neither standard horizontal saccade tests nor standard manual reaction time tests revealed large differences, as shown by the low proportions of patients scoring outside normative ranges on the Auditory Reaction Time (ART), Visual Reaction Time (VRT), or Saccades Horizontal tests (Table 5). However, when saccades and manual reaction time were combined within a joint test requiring both responses in conjunction, significant differences in reaction time emerged (Saccades and Reaction Time test, Motor resp R and L button latency, Table 5). Also, in the vertical saccades test, significant differences were seen for latency and for one of the measures of accuracy (Final accuracy grand mean).

Lastly, while standard saccades were not strongly impaired, other tests showed a significantly impaired ability to generate timed, anticipatory saccades (Predictive Saccades, *N* = 24/77, *p* < 0.0009), and saccades going in the opposite direction from presented stimuli (Antisaccades, *N* = 22/76, *p* < 0.0009).

### Predictive metrics

To examine the ability of our testing protocol to identify PASC in a patient population, we constructed several logistic regression models. Some of these were simple regressions of single variables (univariate), followed by a more complex model using multiple metrics as combined predictors of impairment (multivariate). We constructed the models using our combined subject cohorts (*n* = 77) and the controls (*n* = 300) from the normative database. As a measure of discriminability, for each model we computed the area under the Receiver Operating Characteristics (ROC) curve (AUC).

Univariate analyses identified 11 metrics that could discriminate between controls (healthy subjects) and post-COVID status (Table 6). These metrics largely agreed with the normative range comparison results (Table 5). As shown in Table 6, high-speed OKN gain was strongly predictive of post-COVID subject status (AUC = 0.82). For both horizontal and vertical slow and fast smooth pursuit, the amount of aberrant saccadic activity was more moderately but still significantly related to post-COVID subject status (AUC values ranging from 0.64 to 0.73). Predictive saccade metrics were also significantly predictive (AUC = 0.77 for our two predicted percent measures), as were antisaccade metrics (e.g., AUC = 0.69 for error percent). One notable difference from our normative range comparison results was that univariate models for horizontal saccade metrics were significantly predictive of subject status with, for example, an AUC of 0.79 for one measure of saccade hypometria (final accuracy % of undershoot, see Table 6), and an AUC of 0.68 for latency. This latency result suggests a correlation between saccadic timing and post-COVID health status, which is significant, but which is not revealed by comparison to normative thresholds alone.

The multivariate model was constructed using stepwise logistic regression (Table 7). The analysis identified six metrics from different tests as significant indicators of post-COVID subjects. Together, the AUC was 0.89, the estimated specificity was 98% (with cutoff value of 0.5) and the sensitivity was 88%.

**Table 7:** Multivariate logistic regression results and corresponding accuracy measures.

**Test**	**Metrics**	**ML estimates**		**Diagnostic accuracy**	
		**Estimate**	***p-*value**	**Statistic**	**Rate**
Intercept		5.469	0.001	*AUC*	0.89
Smooth Pursuit Horizontal, 0.75 Hz	Velocity saccade, %	0.078	0.002	*Somers*' *D*	0.79
Smooth Pursuit Vertical, 0.1 Hz	Velocity saccade, %	0.078	0.014	*Accuracy*	0.96
Optokinetic Nystagmus, 60 deg/s	Average gain	−15.600	0.000	*Sensitivity*	0.88
Predictive Saccades	% of predicted	−0.142	0.000	*Specificity*	0.98
Antisaccades	Overall prosaccade % error	0.074	0.001		
Antisaccades	Accuracy grand mean	0.014	0.008		
Number of observations (*Healthy*/*post-COVID*)		375 (*300*/*75*)			

The model was generated using data from 300 normative data subjects and 75 post-COVID patients. Table entries are estimated values of the regression coefficients (ML estimates) with corresponding p-value.

### Neurobehavioral symptom inventory

**Figure 3:**
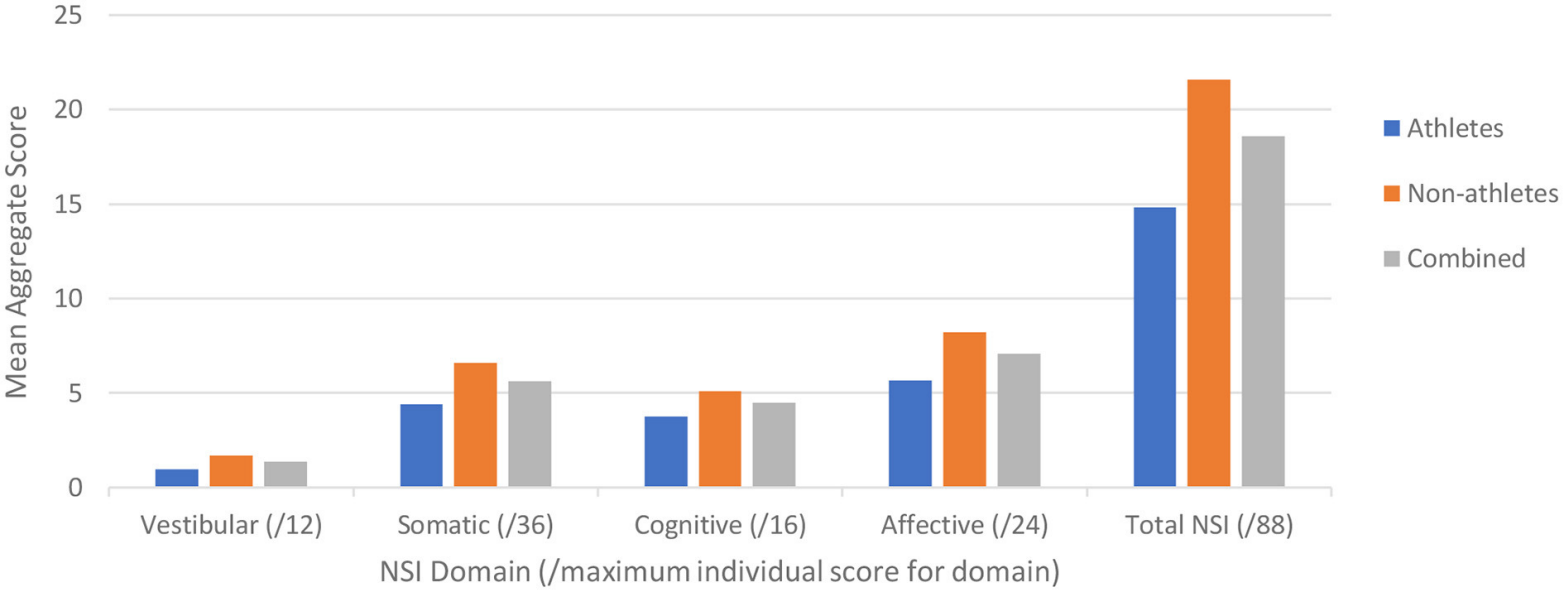
Neurobehavioral Symptom Inventory (NSI) scores plotted for the two cohorts (blue – Cohort 1, orange – Cohort 2, gray – combined) for each NSI domain as identified by Vanderploeg et al. (36), with the maximum score for each domain in the respective parentheses.

Of the 77 participants, 64 completed the NSI. For each NSI domain (i.e., vestibular, somatic, cognitive, and affective), we determined the maximum aggregate score per subject for each domain and overall (e.g., as the maximum score for each NSI item is 4, the aggregate score for each domain is 4^*^(number of NSI items in domain). We then calculated the mean score per domain for each cohort (athletes, non-athletes) and overall (pooled) (Figure 3).

We then examined the relationship between symptom scores and OVRT-C metrics (Table 8). There were moderate but significant correlations between NSI domain key variables and OVRT-C tests. For example, the NSI cognitive domain correlated with Antisaccade errors (*Decisions*, rho = 0.36, *p* = 0.003), Vertical Saccades accuracy (*Forgetfulness*, rho = −0.33, *p* = 0.001), and Self-Paced Saccade rate (*Decisions*, rho = −0.36, *p* = 0.004). Vertical saccade accuracy was also correlated modestly with the NSI vestibular domain *Coordination* variable (% saccade undershoot, rho = 0.39, *p* = 0.001). Spontaneous nystagmus showed correlation with two cognitive domain measures, namely *Forgetfulness* (rho = −0.41, *p* = 0.002) and *Concentration* (e.g., for average slow phase velocity, rho = −0.53, *p* = 0.002).

**Table 8:** Correlation between the NSI domains and OVRT-C tests.

**Test**	**Metrics**	***N***	**rho**	***p*-value**
**(a) Vestibular domain symptoms**				
		*Dizzy*		
Spontaneous nystagmus, in dark	Nystagmus beats, ASPV	48	0.30	0.035
Saccade vertical	Latency grand mean	64	0.27	0.029
Smooth pursuit vertical, 0.1 Hz	Velocity gain up	64	−0.33	0.009
		*Balance*		
Gaze horizontal, in dark	Number of SWJ	35	−0.37	0.029
Smooth pursuit vertical, 0.1 Hz	Velocity gain up	64	−0.29	0.020
		*Coordination*		
Saccade vertical	Accuracy means	64	−0.37	0.002
	Accuracy % of undershoot	64	0.39	0.001
Vergence pursuit	Left/right eye position asymmetry	64	−0.35	0.005
	Near point asymmetry	64	−0.34	0.006
	Far point asymmetry	64	−0.35	0.005
**(b) Somatic domain symptoms**				
		*Headaches*		
Vergence Step	Left inward saccade, %	64	0.40	0.001
	Right inward saccade, %	64	0.38	0.002
		*Nausea*		
Vergence step	Left inward saccade, %	64	0.32	0.010
	Left outward saccade, %	64	0.28	0.023
	Right inward saccade, %	64	0.30	0.015
	Right outward saccade, %	64	0.33	0.009
Smooth pursuit horizontal, 0.1 Hz	Number of SWJ during left	64	0.33	0.008
	Number of SWJ during right	64	0.37	0.003
	Number of SWJ	64	0.39	0.001
Vergence pursuit	Saccadic component, %	64	0.30	0.015
	Saccade move (left), %	64	0.30	0.018
	Saccade move (right), %	64	0.28	0.027
Smooth Pursuit Horizontal, 0.1 Hz	Velocity saccade, %	64	0.36	0.003
	Number of SWJ during left	64	0.32	0.009
	Number of SWJ during right	64	0.36	0.003
	Number of SWJ	64	0.39	0.002
		*Vision problem*		
Smooth pursuit horizontal, 0.1 Hz	Number of SWJ during left	64	0.28	0.025
Gaze horizontal, in dark	Nystagmus beats, PSPV	26	−0.41	0.037
		*Light sensitivity*		
Smooth pursuit horizontal, 0.1 Hz	Number of SWJ during left	64	0.30	0.017
	Number of SWJ	64	0.27	0.030
	Velocity saccade, %	64	0.25	0.048
	Initiation latency, msec	64	−0.34	0.005
Gaze horizontal, in dark	Nystagmus beats, ASPV	29	−0.40	0.034
**(c) Cognitive domain symptoms**				
		*Concentration*		
Spontaneous nystagmus, in dark	Nystagmus beats, ASPV	32	−0.53	0.002
	Nystagmus beats, PSPV	31	−0.43	0.002
		*Forgetfulness*		
Spontaneous nystagmus, in dark	Nystagmus beats, PSPV	31	−0.41	0.002
Saccade vertical	Accuracy grand mean	64	−0.33	0.001
		*Decisions*		
Antisaccades	Overall prosaccade errors	64	0.36	0.003
Self-paced saccades	Saccades per second	64	−0.36	0.004
		*Thinking*		
Antisaccades	Overall prosaccade errors	64	0.36	0.018
Self-paced saccades	Saccades per second	64	−0.27	0.031
**(d) Affective domain symptoms**				
		*Fatigue*		
Gaze horizontal, in dark	Number of nystagmus beats	35	0.38	0.023
Saccades vertical	Latency grand mean	64	0.27	0.033
		*Sleep*		
Saccades vertical	(RU) Latency late response, %	64	0.25	0.050
		*Anxiety*		
Gaze horizontal, in dark	Number of nystagmus beats	35	0.42	0.013
		*Depressed*		
Smooth pursuit vertical, 0.1Hz	Initiation latency, msec	64	−0.30	0.018
		*Irritability*		
Vergence pursuit	Saccade component, %	64	0.34	0.016
	Saccade move (left), %	64	0.31	0.013
	Saccade move (right), %	64	0.27	0.031
Smooth pursuit horizontal, 0.1 Hz	Number of SWJ during left	64	0.35	0.004
	Number of SWJ	64	0.31	0.012
Smooth pursuit vertical, 0.1Hz	Number of SWJ during up	64	0.28	0.027
	Number of SWJ	64	0.25	0.049
		*Frustration*		
Smooth pursuit horizontal, 0.75Hz	Number of SWJ during left	64	0.28	0.023
Smooth pursuit vertical, 0.75Hz	Number of SWJ during up	64	0.26	0.041
	Number of SWJ	64	0.27	0.032

Spearman's rank correlation coefficient (rho) is used to measure the relationship between test metrics and symptoms using the CORR function in MATLAB software (The MathWorks, Inc. USA, version R2015b). • p-value is calculated under two-tail hypothesis. For simplicity the following acronyms are used: • ASPV, average slow phase velocity; • PSPV, peak slow phase velocity; • SWJ, square wave jerks.

## Discussion

The SARS-CoV-2 pandemic has brought public and clinical attention to an area of growing concern regarding viral infections, namely that a patient's emerging from the acute phase of infection and illness does not always imply recovery. This was seen, for instance, with the SARS coronavirus infections of 2002–2004 (SARS-CoV) where a sizeable proportion of patients did not recover their pre-infection health status, and instead entered a chronic period of impairment characterized by fatigue, myalgia, chronic pain, and other factors contributing to significant disability (37). COVID-19 infection has demonstrated a similar pattern where, despite some patients showing complete acute recovery, a significant proportion of patients experience ongoing symptoms months after the acute period of infection (21, 22).

It is now clear that neurological issues feature commonly and prominently in the post-infection period for recovered COVID-19 patients. While fatigue is the most reported symptom, this is followed closely in prevalence by patient-reported impairments in concentration, memory issues, problems with mood and emotion, sleep disruption, “brain fog” and other cognitive disruptions, and dizziness (22). Indeed, our NSI data support these findings, with cognitive (e.g., concentration) and affective (e.g., anxiety) complaints being the most prevalent and severe.

The OVRT-C results reported here reinforce the conclusion that COVID-19—in addition to its post-infection sequelae—is a neurological condition as much as it is a respiratory and autonomic one [see (3, 38) for reviews], and is consistent with two recent studies (31, 32). The most important contribution of VOG measurements is that they do not simply report on the state of oculomotor systems, but rather these metrics are quantifiable and objective proxies and biomarkers for the overall state of brain health. Our results reflect clear CNS impairment in recovered COVID-19 patients and provide support for three important hypotheses. First, we observed that subjects demonstrated a diversity of impairments, for instance in smooth pursuit tracking, saccades, and optokinetic nystagmus responses. Each of these reflect different anatomical substrates, which implies COVID-19 infection can chronically affect a broad range of neuroanatomical territory. Second, there was notable heterogeneity in OVRT-C and NSI data across participants, supporting the hypothesis that chronic COVID-19 neurological effects are themselves heterogeneous, leading to an equally diverse presentation of post-infection symptoms. Lastly, the oculomotor dysfunction we observed implies that COVID-19 infection can lead to significant disability well beyond, and perhaps hidden by, the more prevalent complaints of fatigue, cognitive under-performance, and memory impairment.

A critical finding was that logistic regression analysis using six OVRT-C variables across multiple tests demonstrated excellent discrimination between recovered COVID-19 subjects and controls, with a sensitivity of 88% and specificity of 98%. These data suggest that despite the high degree of heterogeneity of our subjects' reported neurological symptoms following recovery, COVID-19 infection resulted in an identifiable and informative collection of specific OVRT-C deficits in our subjects at the group level. The strength of this pattern may vary based on factors such as disease severity, vaccination status, comorbid disorders, and/or patient age; however, recent evidence suggests that even mild COVID-19 infection is associated with structural abnormalities following recovery (33). This model can be used as a quick screening tool of post-COVID patients to identify who might be at risk of PASC/long COVID and could benefit from immediate treatment.

### ME/CFS and syndromes of chronic fatigue

The mechanisms behind neurological effects of COVID-19 infection are unknown. The etiology and symptoms of recovered COVID-19 patients, however, bear a remarkable resemblance to those of another condition; namely, myalgic encephalomyelitis/chronic fatigue syndrome (ME/CFS). Not only do the presenting symptoms strongly overlap, such as fatigue, post-exertional malaise (PEM), cognitive and memory difficulties, autonomic issues, among others [see (39), for review], but it has been noted that cases of ME/CFS are frequently preceded by instances of infection (40). Equally important, post-infectious fatigue syndromes can result from several viruses, such as SARS-CoV (37), the Epstein-Barr virus (EBV), *Coxiella burnetii*, and Ross River virus (41). Evidence is accumulating that post-COVID symptoms and ME/CFS both result from immunological hyperactivation, whereby an overwhelming activation of immune responses during an acute infectious period leaves the immune system chronically damaged, with long-term consequences for multiple systems that are normally dependent upon proper immune system balance (16, 42, 43). Another compatible hypothesis is that both conditions correlate with brainstem dysfunction, e.g., in medullary regions that are normally responsible for respiration, vasoconstriction, and vestibular responses, among many other functions [e.g., (11); see (42) for review]. While the present study did not aim to identify etiological mechanisms or specific impaired neural targets, the similarities between disorders suggest that long COVID may share many physiological mechanisms with, is etiologically related to, or is in fact the same as ME/CFS, which are hypotheses that have been proposed or assumed by a number of researchers familiar with both conditions (39, 44, 45).

Importantly, even if post-COVID sequelae and other fatigue-dominated syndromes are eventually shown to be distinct in some of their physiological details, the results of VOG testing from one condition can still valuably inform the other, particularly with regard to fundamental relationships between fatigue, cognitive impairment, autonomic dysfunction, and quantifiable metrics of detailed brain functioning, such as those that OVRT-C/VOG testing provide (46). This may prove particularly beneficial when attempting to understand virally initiated fatigue syndromes generally. We also believe it worth noting that our results address one of the valuable lessons learned from the clinical history of ME/CFS, namely that objective measurements are a valuable complement to subjective experience in verifying the individual presence of, and even refuting doubts about the reality of, a disorder otherwise defined largely by subjective symptoms (47).

## Data availability statement

The raw data supporting the conclusions of this article will be made available by the authors, without undue reservation.

## Ethics statement

The studies involving human participants were reviewed and approved by Allegheny Health Network Institutional Review Board (IRB#2020-367), University of Sáo Paulo School of Medicine Institutional Review Board (IRB#33787520.4.0000.0068). The patients/participants provided their written informed consent to participate in this study.

## Author contributions

KK and AKi: co-principal investigator, study design, and manuscript review. RAn: co-principal investigator, data interpretation, and neuroclinical evaluation. AKu: manuscript review and project design. RAs and LG: wrote manuscript and study design. AS: screened, recruited, and enrolled patients. LM: tested patients. AA: tested patients and organized data. RS: study design and background research. SB and RT: neuroclinical evaluation. AB: statistical analysis. All authors contributed to the article and approved the submitted version.

## Funding

The authors declare that this study received funding from Neurolign International Inc. The funder was not involved in the study design, collection, analysis, interpretation of data, the writing of this article or the decision to submit it for publication.

## Conflict of interest

Author AKi is the VP Technology Development, employee of, and shareholder of Neurolign USA LLC. Author RAs is an employee of and shareholder of Neurolign USA LLC. Author LG is an employee of Neurolign USA LLC. Author RAn received funding support from Neurolign USA LLC. The remaining authors declare that the research was conducted in the absence of any commercial or financial relationships that could be construed as a potential conflict of interest.

## Publisher's note

All claims expressed in this article are solely those of the authors and do not necessarily represent those of their affiliated organizations, or those of the publisher, the editors and the reviewers. Any product that may be evaluated in this article, or claim that may be made by its manufacturer, is not guaranteed or endorsed by the publisher.
